# pSATdb: a database of mitochondrial common, polymorphic, and unique microsatellites

**DOI:** 10.26508/lsa.202101307

**Published:** 2022-02-18

**Authors:** Sonu Kumar, Ashutosh Singh, Asheesh Shanker

**Affiliations:** 1 Department of Bioinformatics, Central University of South Bihar, Gaya, India; 2 Translational Bioinformatics Lab, Department of Life Sciences, Shiv Nadar University, Greater Noida, India

## Abstract

Microsatellites, also termed as simple sequence repeats, are repetitive tracts in a DNA sequence, typically consisting of one to six nucleotides. These repeats are found in all genomes and play key roles in phylogeny and species identification. Microsatellites are highly polymorphic, and their length may differ from species to species. There are several online resources dedicated to mitochondria; however, comprehensive information is not available about the length variation of mitochondrial microsatellites. Therefore, to explore it between species among a genus, we have developed a database named pSATdb (polymorphic microSATellites database; https://lms.snu.edu.in/pSATdb/). pSATdb contains 28,710 perfect microsatellites identified across 5,976 mitochondrial genome (mt-genome) sequences from 1,576 genera which includes 1,535 (5,846 mt-genome) and 41 (130 mt-genome) genera of Metazoa and Viridiplantae, respectively. pSATdb is the only database which provides genus-wise information about the length variation of mitochondrial microsatellites. Because of the emerging role of microsatellites in genomics studies, the identified common, polymorphic, and unique microsatellites stored in pSATdb will be effectively useful in various studies including genetic diversity, mapping, marker-assisted selection, and comparative population studies.

## Abstract (precis)

The polymorphic microSATellites database (pSATdb) provides information on common, polymorphic, and unique mitochondrial microsatellites.

## Introduction

Mitochondria, often referred to as the “powerhouses of the cell,” are an essential cellular organelle found in eukaryotes. Mitochondria possess its own genome and plays essential roles in cellular respiration (Roger et al, 2017), phylogeny (Kern et al, 2020), and species identification (Yang et al, 2014; Stoeckle & Thaler, 2018). Mitochondrial genome (mt-genome) contains repetitive sequences including microsatellites with varying lengths (Habano et al, 1998).

Microsatellites, also termed, as simple sequence repeats (SSRs), are a repetitive tract in DNA, typically consisting of one to six nucleotides (Tautz & Renz, 1984). Based on the composition of the repeats, microsatellites were categorized as perfect, imperfect, and compound microsatellites. Repeats without interruption are known as perfect microsatellites (e.g., AAAAAAAA), whereas imperfect microsatellites are interrupted by non-repeat nucleotides (e.g., AAAA***T***AAAA). Two or more microsatellites found adjacent to each other or separated by few nucleotides are called compound microsatellites (e.g., AAAAAAAA*TTTTTTTT*; Bachmann et al, 2004). These repeats are found in coding, non-coding, and coding–non-coding regions of both eukaryotic and prokaryotic genomes (Shanker et al, 2007a, 2007b; Kapil et al, 2014; Kabra et al, 2016; Kumar & Shanker, 2018a). Moreover, these repeats have also been reported in organellar genome including mitochondria (Kumar et al, 2014, 2020; Kumar & Shanker, 2020a).

Microsatellites have been widely applied as a powerful genetic/molecular marker because of their abundance, high reproducibility, hypervariability, codominant and multi-allelic nature (Powell et al, 1996; Parida et al, 2009). Consequently, microsatellites were applied for a variety of purposes including genetic, evolutionary, molecular breeding, and phylogenetic studies (Agarwal et al, 2008; Stolle et al, 2013; Deng et al, 2016; Fu et al, 2016). Earlier, studies were conducted to identify microsatellites in mitochondrial genomes of the order Hypnales (Anand et al, 2019), *Aneura pinguis* (Kumar & Shanker, 2020a), and *Orthotrichum* (Kumar et al, 2020).

Recent advances in database development prove to be a useful resource in many scientific studies (Kumar & Shanker, 2018b, 2018c, 2020b) including characterization of microsatellites (Kumar et al, 2014; Kabra et al, 2016). In view of the immense significance of microsatellites, many specialized databases were developed including Cotton Marker Database (Blenda et al, 2006), EuMicroSatdb (Aishwarya et al, 2007), ChloroMitoSSRDB (Sablok et al, 2013), PIPEMicroDB (Sarika et al, 2013), MitoSatPlant (Kumar et al, 2014), CyanoSat (Kabra et al, 2016), PineElm_SSRdb (Chaudhary et al, 2016), and SSRome (Mokhtar & Atia, 2019).

However, available databases do not provide comprehensive information on common, polymorphic (showing length variation), and unique mitochondrial microsatellites (mtSSRs) between each pair of organisms among a genus. Therefore, we have developed a user-friendly database of pre-mined common, polymorphic, and unique mtSSRs named pSATdb (polymorphic microSATellites database). This database provides genus-specific information on common, polymorphic, and unique mtSSRs and can be utilized for various purposes including genetic diversity, phylogenetic analysis, and species identification.

## Results

### Microsatellite data access

**Figure 1.**
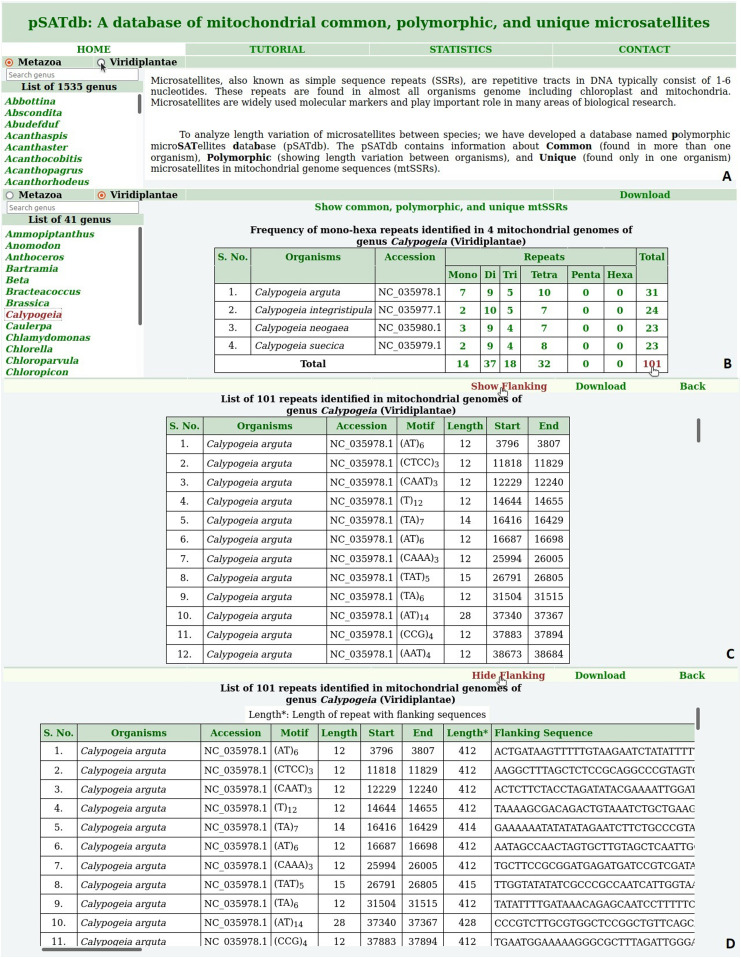
The polymorphic microSATellites database and data access. **(A)** Home page of the polymorphic microSATellites database. **(B)** Mono-/hexa-nucleotide repeats identified in the genus *Calypogeia*. **(C)** Information on repeat motifs in forward (+ve) direction. **(D)** Repeat motifs along with their flanking sequences.

The pSATdb (https://lms.snu.edu.in/pSATdb/) contains genus-wise information on 28,710 perfect microsatellites identified from 5,976 mitochondrial genomes of 1,576 genera which include 1,535 (5,846 mt-genome) and 41 (130 mt-genome) genera of Metazoa and Viridiplantae, respectively. Therefore, the data stored in pSATdb were categorized as Metazoa and Viridiplantae. The framework of pSATdb contains Home, Tutorial, Statistics, and Contact web pages. The “Home” page of the pSATdb provides complete access to the database. A list of genera specific to Metazoa and Viridiplantae can be retrieved by selecting the respective radio buttons. To find the desired genus, a text-based search is also provided (Fig 1A).

The frequency of repeats identified in mt-genome sequences of a genus can be fetched in a tabular form by clicking on the genus name. It will retrieve the frequency of mono-/hexa-nucleotide repeats identified in each mt-genome sequence of the selected genus (Fig 1B). Moreover, various details including repeat motif, length, and start-end position can be fetched by clicking on the respective frequency (Fig 1C). Additionally, flanking sequences of selected repeats can also be retrieved (Fig 1D).

**Figure 2.**
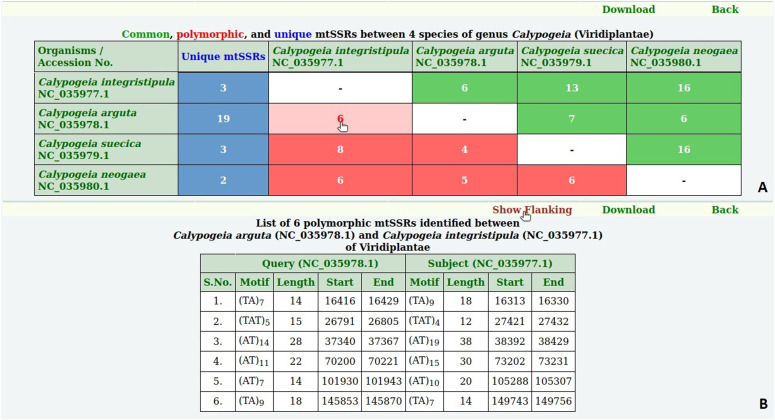
Length variation in polymorphic microSATellites database. **(A)** Summary of common, polymorphic, and unique microsatellites identified in the genus *Calypogeia*. **(B)** Information on polymorphic microsatellites.

Common, polymorphic, and unique microsatellites of the selected genus can be accessed by clicking the hyperlink “Show common, polymorphic, and unique mtSSRs” (Fig 1B). The common and polymorphic microsatellites identified between each pair of species in the selected genus were represented in the form of a matrix, whereas the total number of unique microsatellites identified in each species of selected genus was also shown in the second column of this matrix (Fig 2A).

The details of common, polymorphic, and unique microsatellites can be fetched by clicking on the respective number. It will display the repeat motif, length, and start–end position of the selected microsatellite (Fig 2B). The data stored in pSATdb can be freely downloaded using the download link.

The “Tutorial” page of pSATdb describes the functionality and interpretation of the available data. The “Statistics” page shows information about the total number of genera and species related to Metazoa and Viridiplantae available in the database. The “Contact” page is for sending any suggestion to the developers of pSATdb.

### Database statistics

**Figure 3.**
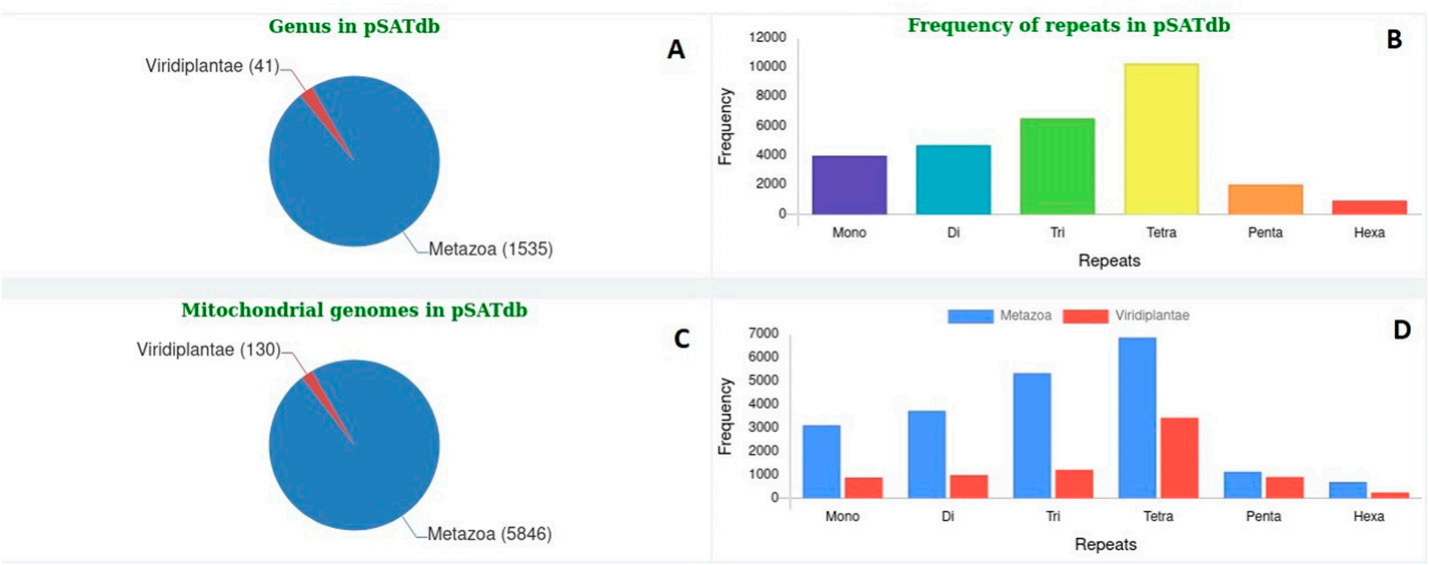
Details of data stored in polymorphic microSATellites database. **(A)** Genera of Metazoa and Viridiplantae available in polymorphic microSATellites database (pSATdb). **(B)** Frequency of mono-/hexa-nucleotide repeats in pSATdb. **(C)** Species of Metazoa and Viridiplantae included in pSATdb. **(D)** Frequency of mono-/hexa-nucleotide repeats in Metazoa and Viridiplantae.

The pSATdb includes 1,535 genera of Metazoa and 41 genera of Viridiplantae (Fig 3A). Among all mt-genome sequences of Metazoa and Viridiplantae considered, tetranucleotides (10,323; 35.96%) were the most prevalent, followed by tri- (6,579; 22.92%), di- (4,750; 16.54%), mono- (4,026; 14.02%), penta- (2,065; 7.19%), and hexa-nucleotide (967; 3.37%) repeats (Fig 3B).

In total, 20,960 microsatellites were identified across 5,846 mt-genomes of Metazoa (Fig 3C). Tetranucleotides (6,875; 32.80%) were the most abundant, followed by tri- (5,354; 25.54%), di- (3,745; 17.87%), mono- (3,131; 14.94%), penta- (1,146; 5.47%), and hexa-nucleotide (709; 3.38%) repeats (Fig 3D).

From 130 mt-genomes of Viridiplantae (Fig 3C), a total of 7,750 microsatellites were identified, with highest frequencies of tetranucleotides (3,448; 44.49%), followed by tri- (1,225; 15.81%), di- (1,005; 12.97%), penta- (919; 11.90%), mono- (895; 11.55%), and hexa-nucleotide (258; 3.33%) repeats (Fig 3D).

Common microsatellites were frequently identified between mt-genomes of closely related species (same genus). The mined data indicated that identified common, polymorphic, and unique microsatellites were not evenly distributed because of the mitochondrial genome composition and size in genera of both Metazoa and Viridiplantae (Table 1).

**Table 1.** Total number of genomes containing common, polymorphic, and unique microsatellites.

Genus (mt-genomes)	Common	Polymorphic	Unique
Metazoa			
536 (1,441)	—	—	✔
460 (1,907)	✔	-	✔
271 (1,627)	✔	✔	✔
100 (312)	✔	—	—
70 (177)	—	—	—
58 (209)	—	✔	✔
29 (149)	✔	✔	—
11 (24)	—	✔	—
Viridiplantae			
24 (84)	✔	✔	✔
10 (30)	—	—	✔
5 (12)	✔	—	✔
2 (4)	✔	✔	—

## Discussion

In this study, microsatellites were identified in mitochondrial genomes of Metazoa and Viridiplantae and further categorized based on their genus as common, polymorphic, and unique. Earlier, mtSSRs were identified in various plants including order Hypnales (Anand et al, 2019), *Aneura pinguis* (L.) Dumort (Kumar & Shanker, 2020a), and *Orthotrichum* (Kumar et al, 2020). Apart from these, SSRs were also mined in chloroplast genomes of *Arabidopsis* (Kumar & Shanker, 2018a) and *Nymphaea* (Kumar & Shanker, 2020c). In all these studies, the distribution of mono-/hexa-nucleotide repeat motifs also varied from species to species, which is congruent with the present study. Earlier, Kumar et al (2014) observed abundance of tetranucleotide repeats in 92 organisms of Viridiplantae, and results of the present analysis are consistent with it.

Common, polymorphic, and unique mtSSRs identified in this study were not equally distributed among each genus of Metazoa and Viridiplantae. The findings are also in harmony with the length variation of microsatellites detected between each pair of species in the genus *Triticum* (Kapil et al, 2014), genus *Arabidopsis* (Kumar & Shanker, 2018a), Order Hypnales (Anand et al, 2019), and genus *Orthotrichum* (Kumar et al, 2020).

Nowadays, information on microsatellites in the public database is growing. Earlier, many databases dedicated to SSRs including Cotton Marker Database (Blenda et al, 2006), EuMicroSatdb (Aishwarya et al, 2007), ChloroMitoSSRDB (Sablok et al, 2013), PIPEMicroDB (Sarika et al, 2013), MitoSatPlant (Kumar et al, 2014), CyanoSat (Kabra et al, 2016), PineElm_SSRdb (Chaudhary et al, 2016), and SSRome (Mokhtar & Atia, 2019) were constructed. However, these databases lack information on common, polymorphic, and unique microsatellites. Therefore, pSATdb was developed to present information on common, polymorphic, and unique microsatellites.

## Materials and Methods

### Data mining

Mitochondrial genome sequences of Metazoa (animals) and Viridiplantae (plants) were downloaded from the National Center for Biotechnology Information in the FASTA file format. Initially, perfect mtSSRs were mined in retrieved mt-genomes with the help of the MIcroSAtellite Identification Tool (https://webblast.ipk-gatersleben.de/misa/; Thiel et al, 2003). The minimum repeat length of ≥12 for mononucleotide, ≥6 for dinucleotide, ≥4 for trinucleotide, and ≥3 for tetra-, penta-, and hexa-nucleotides were considered to mine the microsatellites. Moreover, interruption between two microsatellites was considered as 0.

### Detection of common, polymorphic, and unique mtSSRs

Length variation between mined mtSSRs was detected using in-house–developed Perl scripts. A reciprocal similarity search was performed using the Basic Local Alignment Search Tool (Altschul et al, 1997) to establish homologous relationship between sequences containing mtSSR and, 200 base pairs of flanking sequences from both upstream and downstream of microsatellites or all nucleotides if <200 (Kabra et al, 2016; Kumar & Shanker, 2018a; Kumar et al, 2020). Microsatellites having identical repeating units with equal length and showing significant sequence similarity were categorized as common mtSSRs (found in more than one organism), whereas identical repeating units with unequal length and showing significant sequence similarity were categorized as polymorphic mtSSRs (showing length variation between organisms of a genus).

**Figure 4.**
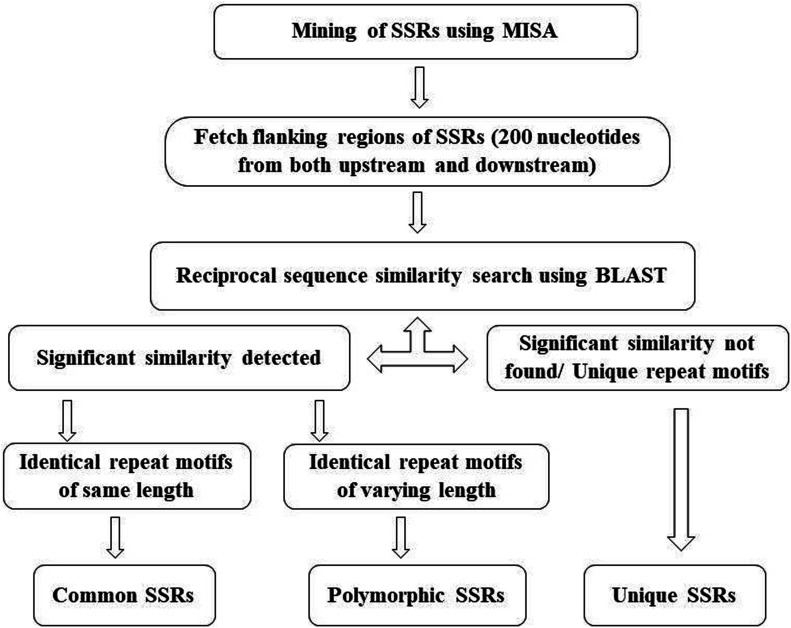
Schematic representation to identify common, polymorphic, and unique microsatellites.

Other repeat motifs and identical repeat motifs showing no significant similarity of flanking sequences with any of the species in the same genus were considered as unique microsatellites (Kumar & Shanker, 2020a, 2020c). A schematic representation to detect common, polymorphic, and unique microsatellites is presented in Fig 4.

### Database development

The pSATdb is a relational database developed using MySQL (v5.5.62). The user interface was designed in HyperText Markup Language along with Cascading Style Sheets, which were used to add style to the database. In the backend, PHP, JavaScript, and AJAX were used. Moreover, JavaScript library CanvasJS and Chart.js were used to generate the graphs.

### Conclusion

A user-friendly, comprehensive database of mitochondrial microsatellites named pSATdb was successfully developed for Metazoa and Viridiplantae. It will act as a ready reference to know the length variation of repeats along with common and unique mitochondrial microsatellites within a genus. We hope that pSATdb will aid researchers working in related fields including molecular marker development, species identification, sequence-tagged sites mapping based on mitochondrial microsatellites.

## Data Availability

The data available in pSATdb (https://lms.snu.edu.in/pSATdb/) are freely accessible/downloadable.

## Supplementary Material

Reviewer comments
